# Unexpected Cardiotocographic Deterioration in Fetal Transient Abnormal Myelopoiesis

**DOI:** 10.7759/cureus.116943

**Published:** 2026-09-25

**Authors:** Kiyoshi Tanaka, Munekage Yamaguchi, Jun Sakata, Takeshi Motohara, Eiji Kondoh

**Affiliations:** 1 Department of Obstetrics and Gynecology, Faculty of Life Science, Kumamoto University, Kumamoto, JPN; 2 Division of Obstetrics and Gynecology, Fukuda Hospital, Kumamoto, JPN; 3 Department of Obstetrics and Gynecology, Hyogo Medical University, Nishinomiya, JPN

**Keywords:** cardiotocographic deterioration, cardiotocography, fetal compromise, fetal transient abnormal myelopoiesis, placental pathology, trisomy 21, umbilical cord pathology

## Abstract

Fetal transient abnormal myelopoiesis (TAM) can rarely occur without prenatal ultrasonographic abnormalities. We describe a case in which fetal compromise was first detected by cardiotocography (CTG). A previously uncomplicated pregnancy showed normal fetal growth and no structural abnormalities on serial ultrasonography. At 38+1 weeks of gestation, CTG revealed markedly reduced baseline variability with recurrent late decelerations, prompting emergency cesarean delivery. The male neonate, who had features of trisomy 21, exhibited marked leukocytosis with circulating blasts. Placental examination revealed intravascular blasts within both chorionic villous and umbilical cord vessels, confirming fetal TAM. Although peripheral blasts initially regressed, acute megakaryoblastic leukemia developed during infancy and required chemotherapy. Fetal TAM may occur without characteristic prenatal sonographic findings, and sudden CTG deterioration may be the first sign of fetal compromise. Placental and umbilical cord pathology can provide crucial postnatal evidence of in utero hematologic disease.

## Introduction

Transient abnormal myelopoiesis (TAM), also known as transient leukemia or transient myeloproliferative disorder, is a unique clonal myeloproliferative disorder that occurs almost exclusively in fetuses and neonates with trisomy 21 [1]. It is characterized by circulating blasts, predominantly of megakaryoblastic lineage, and is closely associated with acquired GATA1 mutations [1]. Approximately 10% of neonates with trisomy 21 develop clinically overt TAM, whereas a larger proportion harbor GATA1-mutated clones with variable hematologic involvement [2]. Although TAM usually resolves spontaneously, approximately 13%-33% of children with clinically overt TAM subsequently develop myeloid leukemia of Down syndrome (ML-DS) within the first four years of life [3].

In contrast to postnatal TAM, fetal TAM is rare and often severe, with approximately 80% of reported cases occurring in the third trimester [4,5]. Reported prenatal findings include hepatosplenomegaly, ascites, hydrops fetalis, pericardial effusion, cardiomegaly, and ventriculomegaly [4,5]. These findings are non-specific and overlap with congenital infections, fetal anemia, metabolic disorders, and hepatic tumors [6]. Fetal TAM is associated with a particularly poor prognosis, especially in cases complicated by both hydrops fetalis and hepatomegaly [5]. In addition to prenatal imaging, fetal TAM has occasionally been diagnosed by umbilical cord blood sampling, and in other cases diagnosis has been supported by placental or umbilical cord pathology, particularly when postnatal hematologic evaluation is limited [7-9]. Notably, intravascular accumulation of myeloblasts within placental and umbilical vessels represents a distinctive histopathologic hallmark of fetal TAM.

In this report, we describe a rare case of fetal TAM in which no prenatal ultrasonographic abnormalities were detected; fetal compromise was first recognized by a sudden deterioration in cardiotocography (CTG), and trisomy 21 was subsequently confirmed postnatally, with the diagnosis further supported by placental pathology.

## Case presentation

A 34-year-old Japanese woman (gravida 3, para 1) with type 1 diabetes mellitus since the age of 11 conceived after ovulation induction. She had a history of cesarean delivery for cephalopelvic disproportion, delivering a 4,630-g female infant. During the current pregnancy, she was treated for Graves’ disease, and both glycemic control and thyroid function remained clinically stable under appropriate medication. Serial fetal ultrasounds demonstrated normal fetal growth with no structural abnormalities, and the prenatal course was uneventful.

At 37+2 weeks of gestation, routine cardiotocography performed during an outpatient antenatal checkup showed a reactive fetal heart rate pattern with normal baseline variability (Figure 1A). A repeat cesarean section was scheduled for 38+2 weeks because of the previous cesarean delivery. On the day prior to the scheduled surgery, CTG performed after admission revealed markedly decreased variability and late decelerations, indicating non-reassuring fetal status (Figure 1B). Ultrasound Doppler examination demonstrated absent end-diastolic flow of the umbilical artery. An emergency cesarean section was, therefore, performed, although the underlying cause of the fetal compromise was unknown.

**Figure 1:**
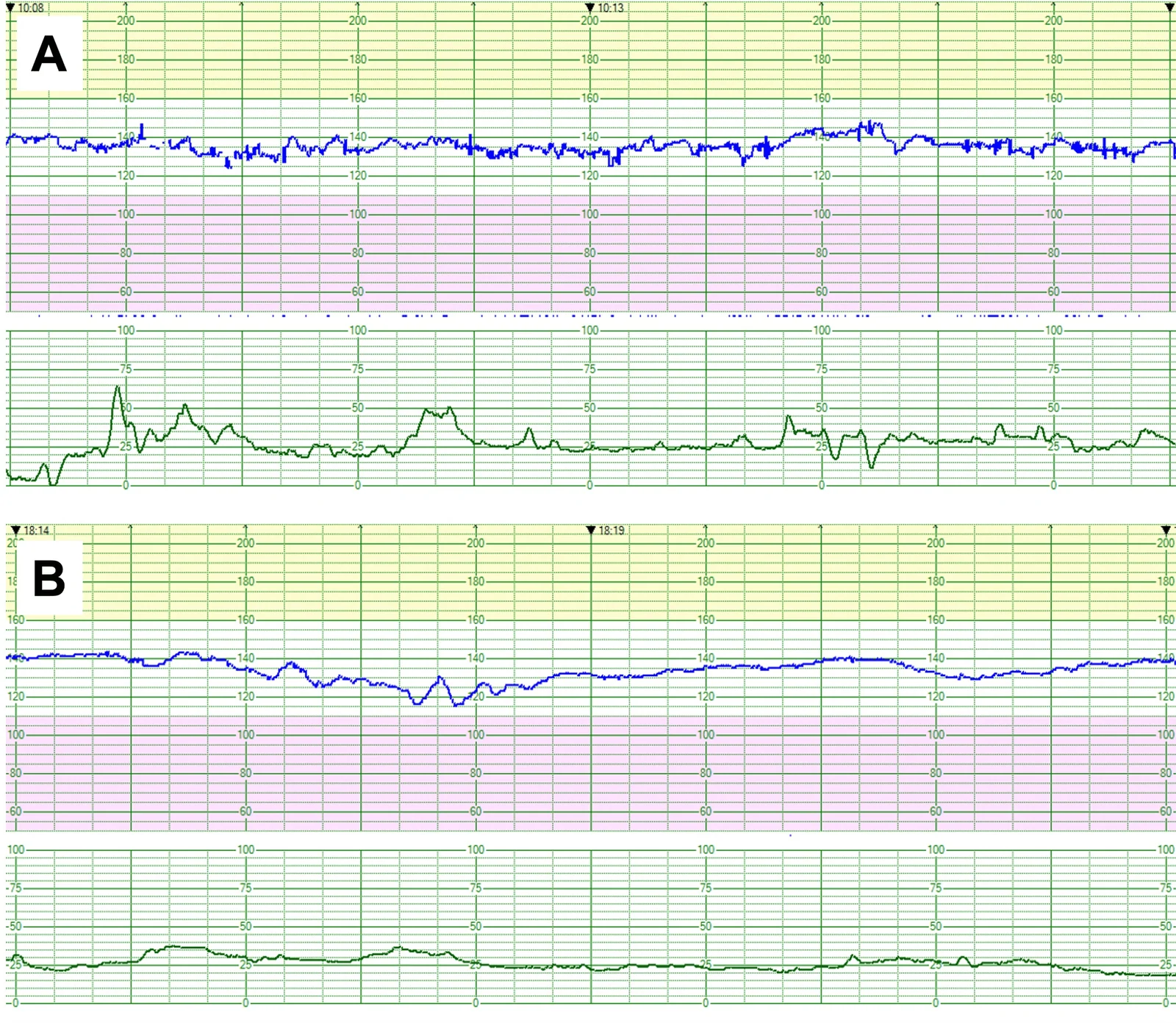
Cardiotocography findings. (A) Cardiotocography at 37+2 weeks of gestation showing a baseline fetal heart rate of 135 beats per minute (bpm) with moderate baseline variability and accelerations, without decelerations. (B) Cardiotocography at 38+1 weeks of gestation demonstrating a baseline of 140 bpm with reduced baseline variability, absence of accelerations, and recurrent late decelerations, indicating non-reassuring fetal status.

A male infant weighing 3,569 g was delivered with Apgar scores of 4 and 7 at 1 and 5 minutes, respectively. Immediately after birth, the infant developed respiratory distress, required ventilatory support with endotracheal intubation, and was admitted to the neonatal intensive care unit (NICU). Physical examination revealed dysmorphic features, including a flat nasal bridge, loose skin at the back of the neck, a single transverse palmar crease, and incurving of the fifth finger, all suggestive of trisomy 21.

Gross placental examination showed a weight of 446 g (approximately the 10th percentile for gestational age). The umbilical cord measured 19 cm in length and contained three vessels. Histopathological examination of the placenta revealed numerous mononuclear cells with a high nuclear-to-cytoplasmic ratio, consistent with blasts, within the vascular lumina of the chorionic villi and within both arteries and veins at the placental insertion site of the umbilical cord (Figure 2). This distribution indicated widespread intravascular fetal hematologic involvement rather than localized placental infiltration.

**Figure 2:**
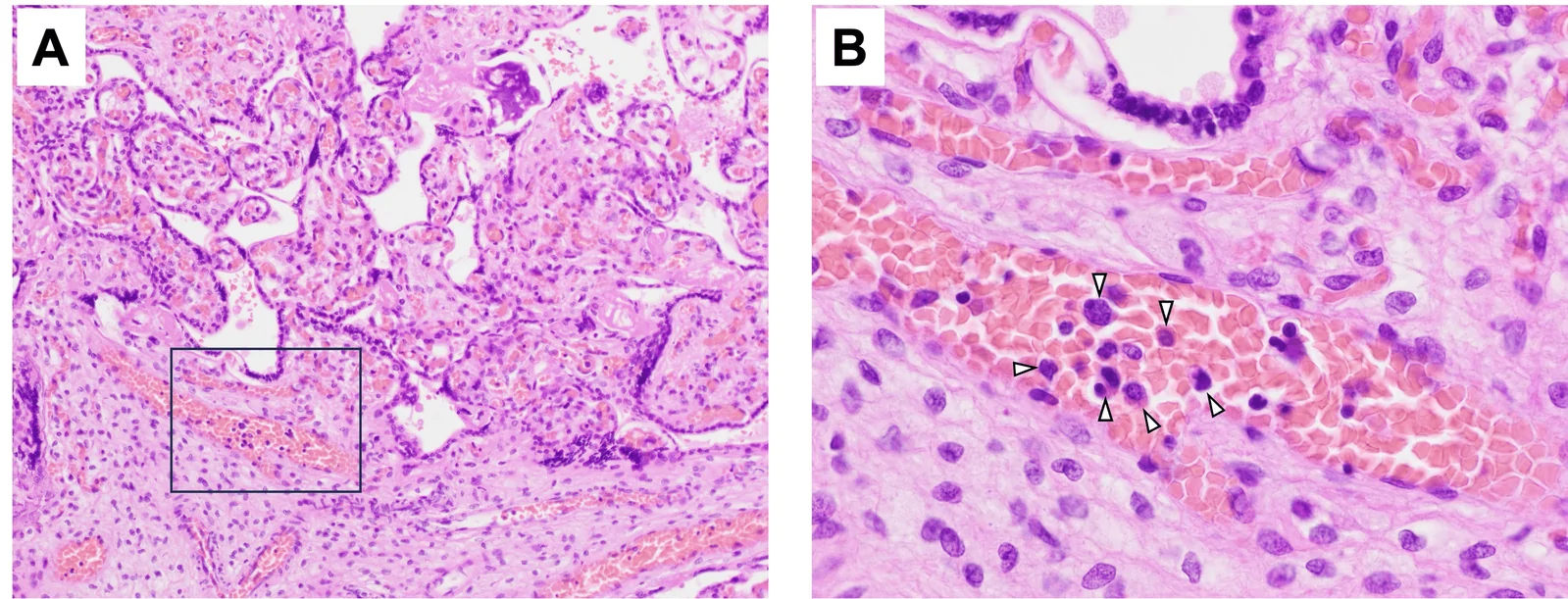
Histopathological findings of the placenta (hematoxylin–eosin staining). (A) Low-power view showing fetal vessels within the chorionic villi and both arteries and veins at the placental insertion site of the umbilical cord. The boxed area corresponds to the higher-magnification image shown in panel B (×20). (B) High-power view demonstrating numerous intravascular mononuclear cells with a high nuclear-to-cytoplasmic ratio consistent with blasts (arrowheads) within the vessel lumina (×40).

Immunohistochemically, these mononuclear cells were diffusely positive for myeloperoxidase (MPO) and largely positive for CD33. CD34 showed granular staining in a subset of these cells (Figure 3). These findings suggested the presence of TAM already developed during the fetal period.

**Figure 3:**
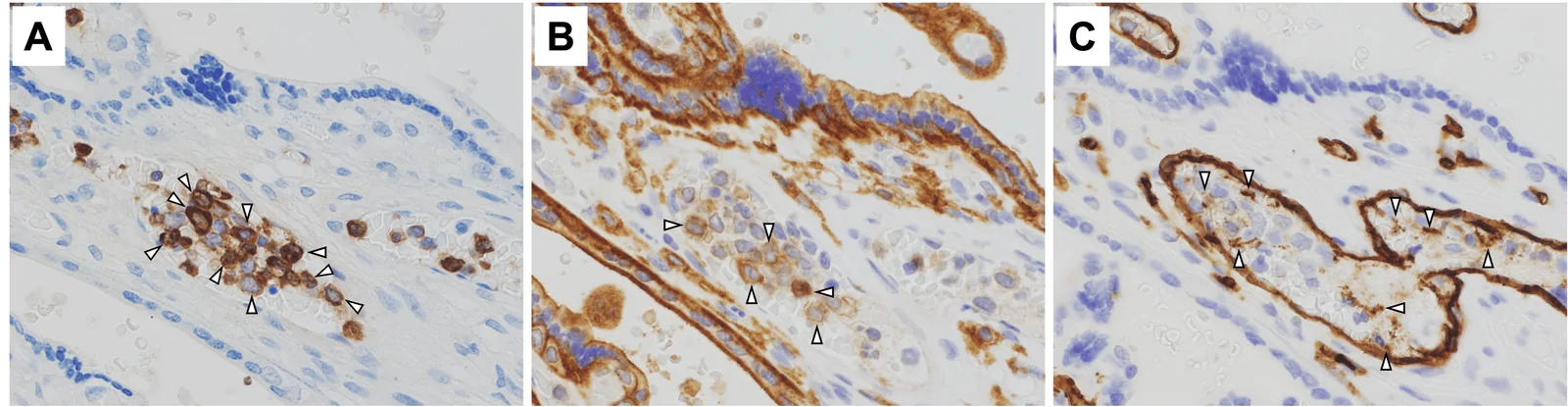
Immunohistochemical analysis of placental vessels. (A) Myeloperoxidase (MPO) staining showing diffuse positivity in the intravascular mononuclear cells. (B) CD33 staining demonstrating positivity in the majority of the intravascular mononuclear cells. (C) CD34 staining showing granular positivity in a subset of these cells.

Postnatal laboratory evaluation demonstrated marked leukocytosis, with a white blood cell count of 65,100/μL (institutional reference range: 3,300-8,600/μL) and circulating blasts accounting for 49% of peripheral leukocytes. The blast proportion increased to 55% on day seven of life; however, it subsequently declined spontaneously, decreasing to 16% by day 13. Chromosomal analysis using G-banding was performed on day 22 and confirmed trisomy 21. By day 50 of life, the blasts had completely disappeared.

At one year and four months of age, follow-up hematologic testing again revealed approximately 5% circulating blasts. Bone marrow examination demonstrated a significant population of immature myeloid cells, and immunohistochemical analysis showed CD42a positivity in 10%-15% of nucleated cells. These findings were consistent with acute megakaryoblastic leukemia (AMKL; FAB M7). Chemotherapy with the AMLD-CET regimen (cytarabine, etoposide, and pirarubicin) was initiated at one year and six months of age. Complete remission was confirmed on bone marrow evaluation at one year and eight months, and the patient was categorized as standard risk. Consolidation therapy (S-1 to S-4) was subsequently completed, and the patient was discharged home at two years of age.

## Discussion

TAM occurs in approximately 10% of neonates with trisomy 21 and is closely linked to acquired GATA1 mutations in fetal and neonatal hematopoietic cells [2]. Although circulating blasts are frequently observed in neonates with trisomy 21, a blast proportion of ≥10% is generally regarded in clinical practice as a practical threshold for the diagnosis of TAM [10]. No universally accepted blast-percentage threshold exists; diagnosis relies on a combination of hematologic abnormalities, clinical findings, and cytogenetic or molecular confirmation [11]. In the present case, the presence of blasts reaching 49% was first identified on postnatal blood testing, leading to a diagnosis of TAM. Subsequent placental pathology confirmed that TAM had already developed during the fetal period. However, from a prenatal perspective, the fetal condition appeared clinically unremarkable until sudden deterioration occurred.

Most previously reported fetal TAM cases have been detected by ultrasound, with hepatosplenomegaly being the most frequent and predictive finding [5]. Hepatomegaly is thought to reflect blast infiltration, hepatic fibrosis, and portal hypertension [12]. Additional features include ascites, hydrops fetalis, pericardial effusion, cardiomegaly, and ventriculomegaly [4,5]. Fetal TAM is associated with poor outcomes, and an overall survival rate of less than 40% has been reported, particularly in cases complicated by hydrops fetalis; similarly high mortality rates have been described, with all fatal cases accompanied by hydrops [4,5]. Nevertheless, these sonographic abnormalities are non-specific, and even in the presence of underlying hematologic disease, some fetuses may, as in the present case, exhibit only minimal ultrasound abnormalities or none at all [6].

Several reports have demonstrated that umbilical cord blood sampling can allow prenatal or perinatal diagnosis of fetal TAM. In trisomy 21 fetuses presenting with TAM-related findings such as hepatomegaly or hydrops, fetal blood sampling frequently demonstrates hematologic abnormalities including leukocytosis and circulating blasts, which are associated with adverse perinatal outcomes [7]. These observations indicate that fetal blood analysis can be valuable when sonographic abnormalities raise suspicion of an underlying hematologic disorder. In addition, placental and umbilical cord pathology has been recognized as an important diagnostic adjunct in fetal TAM. Previous studies have shown that myeloblasts can be identified within the vascular lumina of the placenta, including the chorionic villi and chorionic plate, as well as within umbilical cord vessels, and that these intravascular blasts are immunoreactive for myeloid markers such as CD34, CD61, and MPO [8, 9]. These lesions reflect circulating fetal leukemic cells rather than placental infiltration, supporting their value as a surrogate of in utero hematologic disease. Such pathological findings are considered characteristic of TAM associated with trisomy 21 and may provide crucial diagnostic information, particularly in cases of stillbirth or early neonatal death in which postnatal hematologic evaluation is limited. In contrast to these previously reported cases, our patient showed no prenatal sonographic abnormalities and did not undergo fetal blood sampling. Instead, acute fetal compromise was detected only by CTG, and placental and umbilical vascular pathology obtained after delivery provided important evidence supporting the diagnosis of fetal TAM.

Favorable treatment outcomes have been reported in children with ML-DS. A reduced-intensity chemotherapy regimen consisting of cytarabine, etoposide, and pirarubicin, the same three agents used in the present case, achieved a four-year event-free survival rate of 83% in 72 children [13]. Similarly, the Children's Oncology Group AAML0431 trial, which employed a multiagent chemotherapy regimen incorporating cytarabine, daunorubicin, thioguanine, L-asparaginase, and etoposide, reported five-year overall and event-free survival rates of 93.0% and 89.9%, respectively [14].

Prenatal recognition of trisomy 21 is frequently missed in routine obstetric care, as ultrasound alone fails to detect more than one-third of affected fetuses [15,16]; moreover, fetal TAM lacking characteristic ultrasonographic abnormalities is exceedingly difficult to diagnose antenatally. Had delivery been delayed in the present case, the risk of intrauterine fetal demise might have been considerable. This is the first report to document a sudden, time-dependent deterioration on CTG in a fetus subsequently diagnosed with TAM. This observation suggests that functional fetal surveillance by CTG may detect acute fetal compromise associated with hematologic or circulatory deterioration even when structural abnormalities are absent on ultrasonography.

## Conclusions

Fetal TAM may cause acute fetal compromise even in the absence of characteristic prenatal ultrasonographic abnormalities. Sudden deterioration on CTG may be the first indication of fetal compromise, highlighting the potential value of functional fetal surveillance when structural abnormalities are absent. Placental and umbilical cord pathology can provide important postnatal evidence of in utero hematologic disease and may help establish the diagnosis of fetal TAM when antenatal recognition is difficult.

## References

[1] Roy A, Roberts I, Vyas P (2012). Biology and management of transient abnormal myelopoiesis (TAM) in children with Down syndrome. Semin Fetal Neonatal Med.

[2] Webb D, Roberts I, Vyas P (2007). Haematology of Down syndrome. Arch Dis Child Fetal Neonatal Ed.

[3] Klusmann JH, Creutzig U, Zimmermann M (2008). Treatment and prognostic impact of transient leukemia in neonates with Down syndrome. Blood.

[4] Robertson M, De Jong G, Mansvelt E (2003). Prenatal diagnosis of congenital leukemia in a fetus at 25 weeks' gestation with Down syndrome: case report and review of the literature. Ultrasound Obstet Gynecol.

[5] Tamblyn JA, Norton A, Spurgeon L (2016). Prenatal therapy in transient abnormal myelopoiesis: a systematic review. Arch Dis Child Fetal Neonatal Ed.

[6] Smrcek JM, Baschat AA, Germer U, Gloeckner-Hofmann K, Gembruch U (2001). Fetal hydrops and hepatosplenomegaly in the second half of pregnancy: a sign of myeloproliferative disorder in fetuses with trisomy 21. Ultrasound Obstet Gynecol.

[7] Hojo S, Tsukimori K, Kitade S, Nakanami N, Hikino S, Hara T, Wake N (2007). Prenatal sonographic findings and hematological abnormalities in fetuses with transient abnormal myelopoiesis with Down syndrome. Prenat Diagn.

[8] Kuo E, Kumarapeli AR (2020). Placental pathology in Down syndrome-associated transient abnormal myelopoiesis. Arch Pathol Lab Med.

[9] Yamazaki S, Miyauchi J, Sato H, Takahashi Y, Amikura T (2024). Autopsy case of a stillbirth with transient abnormal myelopoiesis associated with Down syndrome: immunohistochemical demonstration of GATA1 mutation in placental tissues. Pathol Int.

[10] Bhatnagar N, Nizery L, Tunstall O, Vyas P, Roberts I (2016). Transient abnormal myelopoiesis and AML in Down syndrome: an update. Curr Hematol Malig Rep.

[11] Singh A, Mandal A, Guru V, Srinivasan S, Seth R (2017). Transient abnormal myelopoiesis: a varied spectrum of clinical presentation. J Hematol.

[12] Hamada H, Yamada N, Watanabe H, Okuno S, Fujiki Y, Kubo T (2001). Hypoechoic hepatomegaly associated with transient abnormal myelopoiesis provides clues to trisomy 21 in the third-trimester fetus. Ultrasound Obstet Gynecol.

[13] Kudo K, Kojima S, Tabuchi K (2007). Prospective study of a pirarubicin, intermediate-dose cytarabine, and etoposide regimen in children with Down syndrome and acute myeloid leukemia: the Japanese Childhood AML Cooperative Study Group. J Clin Oncol.

[14] Taub JW, Berman JN, Hitzler JK (2017). Improved outcomes for myeloid leukemia of Down syndrome: a report from the Children's Oncology Group AAML0431 trial. Blood.

[15] Papp C, Bán Z, Szigeti Z, Csaba A, Lázár L, Nagy GR, Papp Z (2007). Prenatal sonographic findings in 207 fetuses with trisomy 21. Eur J Obstet Gynecol Reprod Biol.

[16] Parra-Cordero M, Quiroz L, Rencoret G, Pedraza D, Muñoz H, Soto-Chacón E, Miranda-Mendoza I (2007). Screening for trisomy 21 during the routine second-trimester ultrasound examination in an unselected Chilean population. Ultrasound Obstet Gynecol.

